# A robust method to estimate the intracranial volume across MRI field strengths (1.5T and 3T)

**DOI:** 10.1016/j.neuroimage.2010.01.064

**Published:** 2010-05-01

**Authors:** Shiva Keihaninejad, Rolf A. Heckemann, Gianlorenzo Fagiolo, Mark R. Symms, Joseph V. Hajnal, Alexander Hammers, The Alzheimer's Disease Neuroimaging Initiative

**Affiliations:** a Division of Neuroscience and Mental Health, MRC Clinical Sciences Centre, Imperial College London, London, UK; b Neurodis Foundation, CERMEP-Imagerie du Vivant, Lyon, France; c Imaging Sciences Department, MRC Clinical Sciences Centre, Imperial College London, London, UK; d NSE MRI Unit, Department of Clinical and Experimental Epilepsy, Institute of Neurology UCL, Queen Square, London, UK

**Keywords:** Human brain, MRI, Volumetry, Intracranial volume, Tissue probability maps, SPM, Reverse normalization

## Abstract

As population-based studies may obtain images from scanners with different field strengths, a method to normalize regional brain volumes according to intracranial volume (ICV) independent of field strength is needed. We found systematic differences in ICV estimation, tested in a cohort of healthy subjects (*n* = 5) that had been imaged using 1.5T and 3T scanners, and confirmed in two independent cohorts. This was related to systematic differences in the intensity of cerebrospinal fluid (CSF), with higher intensities for CSF located in the ventricles compared with CSF in the cisterns, at 3T versus 1.5T, which could not be removed with three different applied bias correction algorithms. We developed a method based on tissue probability maps in MNI (Montreal Neurological Institute) space and reverse normalization (reverse brain mask, RBM) and validated it against manual ICV measurements. We also compared it with alternative automated ICV estimation methods based on Statistical Parametric Mapping (SPM5) and Brain Extraction Tool (FSL). The proposed RBM method was equivalent to manual ICV normalization with a high intraclass correlation coefficient (ICC = 0.99) and reliable across different field strengths. RBM achieved the best combination of precision and reliability in a group of healthy subjects, a group of patients with Alzheimer's disease (AD) and mild cognitive impairment (MCI) and can be used as a common normalization framework.

## Introduction

The quantification of the sizes of anatomical structures within the human brain is improving due both to advances in magnetic resonance (MR) imaging sequences and to progress in image analysis. Following the realization that variation in the volume of a particular brain structure may be related to variation in head size, a common approach to the problem of variability has been to employ some correction for overall head size (Gold and Squire, 2005). A number of different normalization procedures have been studied, including brain area on a 2D section at a specific level (Gold and Squire, 2005; Galton et al., 2001; Free et al., 1995), cranial volume, and intracranial volume (ICV) (Obenaus et al., 2001; Marsh et al., 1997; Murphy et al., 1992).

Normalization by intracranial volume reduced variability in the volume measurements of nearly all brain regions to a greater extent than did normalization by other methods (Free et al., 1995). ICV has generally been measured by manual delineation of the intracranial portion of volumetric T1-weighted images, a laborious and time consuming procedure requiring the manual tracing of a large region.

In addition to manual approaches, various automated methods have also been used, including Statistical Parametric Mapping (SPM) (Ashburner and Friston, 2005; Ananth et al., 2002; Sanfilipo et al., 2004), Exbrain (Lemieux et al., 2003; Hammers et al., 2007), and the Brain Extraction Tool (BET) (Smith, 2002). (Pengas et al., 2009) compared the reliability of different ICV estimation methods to establish whether such methods are influenced by brain atrophy in semantic dementia. Other studies have compared the performance of different skull-stripping algorithms (Boesen et al., 2004; Rex et al., 2004; Segonne et al., 2004). More recently, some studies succeeded in measuring ICV using atlas-based algorithms via a template warping algorithm modified for head image registration (Buckner et al., 2004; Driscoll et al., 2009).

Practically all of these methods have been using MR images obtained on scanners with a B_0_ field strength of 1.5T. There are few studies estimating ICV on images obtained from 3T scanners (Rex et al., 2004; Suja and Rakesh, 2004). To our knowledge, no study of consistency across field strengths of ICV measurement has previously been conducted. Increasing availability of large-scale datasets acquired on different scanners in longitudinal and multicentre structural neuroimaging studies^2^ makes this a pressing issue.

In this work, we investigate differences in ICV calculated for two types of scanner (1.5T and 3T). The primary objective was to assess the comparability of ICV measurements based on different methods on images of subjects who had been scanned at both field strengths. To reduce the difference between ICV measurements in datasets obtained from scanners with different field strengths, we applied three different bias correction methods. All three failed to eliminate the bias we observed. We therefore implemented an automated method of measuring intracranial volume, Reverse MNI Brain Masking (RBM), based on tissue probability maps in MNI standard space. We validated the new method by comparison with ICV measurements obtained by expert manual outlining on five healthy subjects and two subjects with Alzheimer's disease. Our findings motivated an investigation into the causes of the phenomenon. Measurements of signal intensity in different CSF spaces showed a pronounced intensity difference between intraventricular CSF and subarachnoid CSF in images obtained from 3T scanners. The RBM method was equivalent to manual ICV measurement for both scanners (ICC = 0.99) and achieved higher accuracy in healthy controls as well as in patients with AD. RBM was consistent and reliable for estimating ICV for images obtained at different field strengths (1.5T and 3T).

## Materials and methods

### Experiment overview

**Fig. 1:**
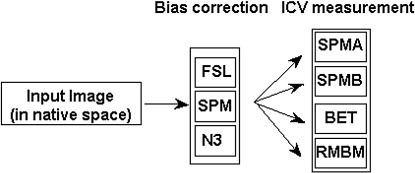
The data processing protocol is exemplified for one input image (the same protocol was used for each of the 10 images acquired from Group 1). Each volume was processed with three bias field correction algorithms (FAST, SPM5, N3) and three ICV measurement algorithms (two parameter sets for SPM, one for BET). See text for further details.

An overview of the analysis strategy is shown in Fig. 1. For the example of Group 1, ten T1-weighted image volumes were acquired from five subjects on 1.5T and 3T scanners. Each image volume was then processed in two steps: (1) non-uniformity correction and (2) ICV measurement. Three publicly available algorithms were used for Step 1. Two publicly available algorithms were used in Step 2, where parameter sets were varied with a view to improving the accuracy of the resulting ICV measurement. The proposed robust method, RBM, was used as a third option in the second step.

For each image volume, the intracranial portion identified through each acquisition/analysis pathway was assessed using the manually segmented intracranial portion as a gold-standard reference. Metrics used for the comparison were the relative volume difference and its magnitude, expressed as a percentage of the average intracranial volume, and spatial overlap, expressed as the Dice coefficient (Dice, 1945). Finally, the influence of scanner field strength on CSF intensities as a potential reason for ICV variations was studied.

### Subjects

Sets of T1-weighted images from three different groups were used in this study.

Group 1: Five healthy volunteers (mean ± SD age of 66 ± 11 years) from the NeuroGrid project. Each subject was scanned on two different magnets, a Philips Achieva 3T unit (Philips Healthcare, Best, the Netherlands) located in the Robert Steiner Unit, Hammersmith Hospital, London, UK, and a GE Signa 1.5T unit (GE Healthcare, Milwaukee, WI, USA) located at the Institute of Neurology, University College London, Queen Square, London, UK. Both scans were acquired within two weeks of each other. 3T images were acquired sagittally with an eight channel SENSE head receive coil using magnetisation prepared fast gradient echo (MP-RAGE), TE/TR 4.6 ms /9.6 ms, time of inversion (TI) 1250 ms, flip angle 8°, yielding 150 slices of 1.2 mm thickness with a field of view of 24 × 24 cm for a 208 × 208 matrix, covering the whole brain with voxels of 1.2 × 1.2 × 1.2 mm^3^. In the case of the 1.5T scanner, a coronal T1-weighted 3D volume was obtained with a birdcage receiver coil using an inversion recovery prepared fast gradient echo, TE/TR 4.2 ms/13 ms, time of inversion (TI) 450 ms, flip angle 15°, yielding 124 slices of 1.5 mm thickness with a field of view of 28 × 28 cm for a 192 × 192 matrix, covering the whole brain with voxel sizes of 1.2 × 1.2 × 1.5 mm^3^.

Group 2: Five subjects diagnosed with Alzheimer's disease (mean ± SD age of 71 ± 11 years) and 10 subjects with mild cognitive impairment (MCI) (mean ± SD age of 67 ± 7 years) from the NeuroGrid project scanned at both field strengths with the same acquisition parameters as Group 1.

Group 3: 10 subjects studied as part of the IXI project, including five subjects scanned at 3T (Philips scanner, as above), and five different subjects scanned at 1.5T (Guy's Hospital).

Group 4: Five subjects from the ADNI project,^3^ who were imaged with various 1.5T (2 × GE, 1 × Philips and 2 × Siemens) and 3T scanners (2 × Philips, 1 × GE, 2 × Siemens) and all had one dataset acquired at 1.5T as well as one acquired at 3T. All images were obtained in pre-processed form (GradWarp, B1 non-uniformity correction and N3 (Sled et al., 1998) applied).

### Non-uniformity correction

MR images are usually degraded by a smooth, spatially varying artifact due to hardware, such as radio frequency (RF) coil non-uniformities, that modulate the intensity of the images. Although these artifacts do not usually interfere with visual inspection, they can adversely affect the performance of downstream processing, such as skull stripping and tissue class segmentation (Simmons et al., 1994; Cohen et al., 2000). Three different algorithms designed to perform bias correction (BC) were used in this study:

*FMRIB*'*s Automated Segmentation Tool v.4.1 (FAST)*: bias field correction provided in the FSL package (Zhang et al., 2001), which incorporates a Hidden Markov Random Fields (HMRF) model and an expectation-maximization (EM) algorithm into a HMRF-EM framework to solve the inhomogeneity problem.

*SPM5 bias correction*: “Unified Segmentation” is a tool that performs simultaneous spatial normalisation, tissue classification and bias correction (Ashburner and Friston, 2005).

*Nonparametric nonuniformity normalization v.1.05 (N3)*: N3 corrects intensity nonuniformities without requiring a tissue class model. It employs a deconvolution kernel to sharpen the intensity histogram plots that have been smoothed by the bias field (Sled et al., 1998).

It is well known that N3 and FAST are more accurate when the brain has previously been segmented from background (Zhang et al., 2001; Sled et al., 1998). Nevertheless, we applied these two bias correction methods directly on the original MR image without applying brain extraction because the main purpose was to measure intracranial volume. Skull stripping may remove parts of the intracranial portion, leading to falsely small ICV results.

### Manual delineation of ICV

**Fig. 2:**
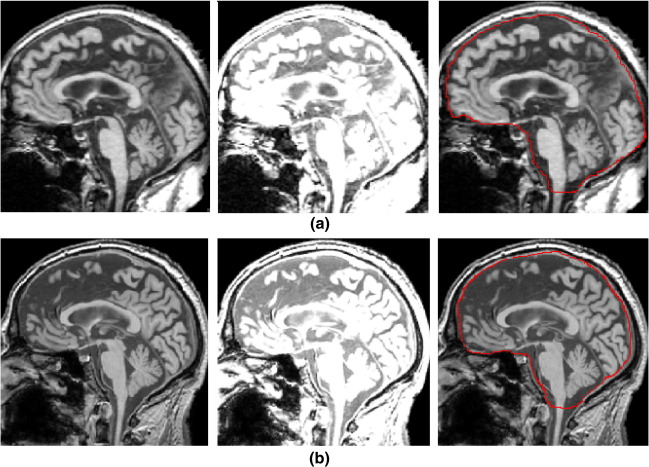
Measurement of the intracranial volume (ICV) for a subject from Group 1 scanned with the 1.5T (a) and 3T (b) scanner. For each group, the original image was reformatted to sagittal sections, which were then magnified by a factor of two. The boundary of the dura mater can not be shown clearly in the initial sagittal image (left). To improve the clarity of the boundary of the dura mater, the brightness of the image was increased (middle). The outer edge of the dura mater was traced by the rater manually. The caudal boundary of the cerebellum was considered the caudal boundary of the intracranial cavity (right).

Manual measurements as the gold standard were performed on T1-weighted images from five healthy controls (Group 1) and two patients with AD (Group 2) using ANALYZE AVW 8.1 software (Biomedical Imaging Resource, Mayo Clinic, Rochester, MN, USA). All delineations were performed by a single observer (SK) and reviewed by a second observer (AH). The recorded time taken to estimate the ICV was approximately 30 min/scan, but no time limit was imposed. Volumetric analysis was performed based on the manual estimation method described in (Chey et al., 2006; Eritaia et al., 2000). The measurements in Group 1 were performed twice at an interval of one month to assess the intra-rater reliability. Details of the measurement of the ICV are illustrated in Fig. 2. In brief, the original slices were reformatted into the sagittal plane. Realigning the original slices to correct for head tilt was not necessary due to the large size of the intracranial cavity (Eritaia et al., 2000). The brightness of the image was increased to improve the visual clarity of the boundary of the dura mater (Chey et al., 2006). In each case, starting from the left-hand side of the head, the slice in which the brain initially appeared was selected as the starting slice and then every tenth slice was included, based on the sampling and accuracy results of Eritaia et al. (2000). The ICV was then estimated for each volume by adding the traced volumes from the segmented slices and multiplying by 10 to obtain the ICV.

### Automated ICV estimation methods

#### SPM tissue-class based method

An estimation of the intracranial portion of an image can be obtained as the sum of three tissue compartments which are provided by SPM5 (Sanfilipo et al., 2004; Valenzuela et al., 2008). A hard cut-off was used in order to exclude any voxel whose probability of belonging to any of the three classes was less than an iteratively determined threshold. In order to calculate the ICV with the SPM-tissue class method, the number of surviving voxels was obtained and multiplied by the volume of a single voxel.

This procedure was applied with two different settings in SPM5: (1) using SPM5 with the default parameters (SPMA), (2) using four Gaussians for CSF classification (SPMB). The rationale for the parameter change in SPMB was the expectation that CSF intensity in 3T images would be better modeled than with the default setting of two Gaussians^4^.

Previous studies suggested that SPM5 is more accurate if it does not attempt to estimate bias fields when nonuniformities are not present (Ashburner and Friston, 2005), therefore in all the experiments in the second step the bias correction was disabled by providing parameter settings that caused a negligible effect over the volume of interest: bias regularisation was set to 10 and bias FWHM was set to 150-mm cut-off (Acosta-Cabronero et al., 2008). Defining the threshold value is an important issue. It should (1) produce a result as close as possible to manual measurement and (2) be compatible and applicable for both scanners with different field of strength. Experiments in this study showed that a suitable threshold value for both scanners using FAST and SPM in the bias correction step and SPMA and SPMB in the ICV measurement step was 90%. In the case of N3 for bias correction we used a threshold value of 50%.

#### Brain extraction tool (BET) in FSL

We also performed BET (FMRIB library,^5^ Oxford, UK) on images of all subjects. In brief, this method uses a surface model approach that starts by finding the centre of gravity and tessellates the brain surface using connected triangles (Clark et al., 2006). When applying BET to the raw images, our results were unsatisfactory due to inclusion of peri-orbital fat, eyes and other non-brain structures in some cases. While other authors have corrected such errors manually (Battaglini et al., 2008), we avoided user intervention by applying standard-space masking (standard_space_roi in FSL 4.1), which removed eye and neck tissue. We then applied BET to the masked image, setting the intensity threshold (‘-f’ parameter) to 0.2 and the vertical gradient of the intensity threshold (‘-g’ parameter) to 0 (flat gradient). These settings were determined by “hand-tuning” using a subset of subjects, then applied to all subjects and the results visually checked.

#### Reverse MNI brain mask method

All subjects' scans were segmented by tissue class with SPM5 (Ashburner and Friston, 2005). The RBM method used the sum of the three prior tissue probability maps without any thresholding to estimate the ICV probabilistic mask in standard space. In SPM5, the probability maps are estimated using a modified version of the ICBM Tissue Probability Maps^6^. The tissue probability maps are originally derived from 452 T1-weighted scans, which were aligned with an atlas space, corrected for scan inhomogeneities, and classified into GM, WM and CSF. These data were then affine registered to the MNI space and downsampled to 2 mm resolution.

**Fig. 3:**
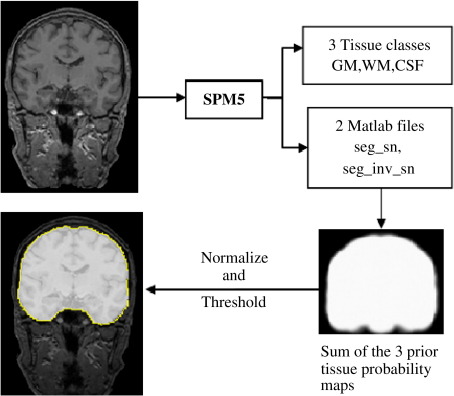
A simplified diagram of the RBM method showing the data flow from a raw MRI to a completed brain mask. The steps involved in this method are tissue class segmentation with SPM5, and warping the sum of the three prior tissue probability maps using the inverted deformation from standard space to subject native space.

The inverted deformation from standard space to subject native space, derived from SPM5′s unified segmentation (Ashburner and Friston, 2005), was used to warp the ICV probabilistic mask in standard space to each image in native space with nearest neighbour interpolation. The inverse normalization was done using SPM5 (Normalise option) and setting the bounding box and voxel sizes to non-finite values. The resulting image was thresholded at 90% probability and the volume of ICV was measured as the number of resulting voxels multiplied by the volume of a single voxel. The processing flow diagram of the RBM method is shown in Fig. 3.

### Influence of field strength on ICV measurement

**Fig. 4:**
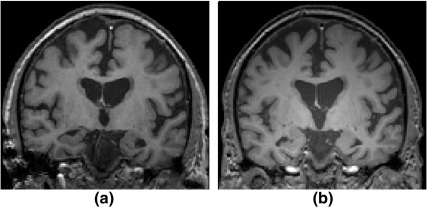
Coronal view of T1-weighted images of a subject from Group 1 scanned at 1.5T (a) and 3T (b). (a) The 1.5T image has a uniform image appearance, (b) The 3T image displays a central brightening artifact.

**Fig. 5:**
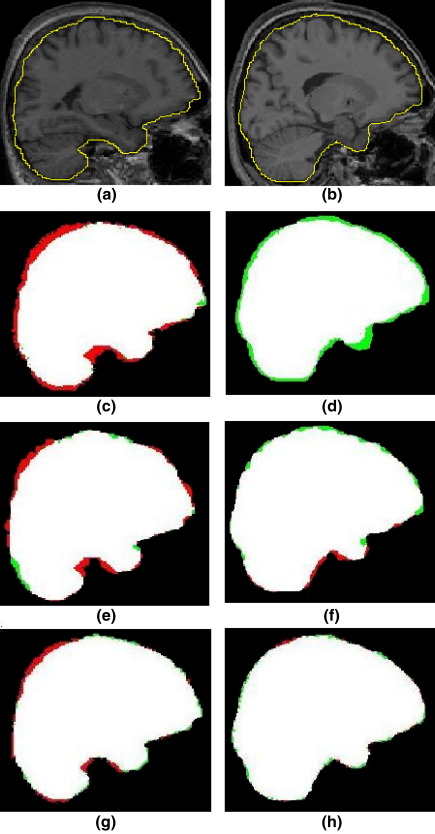
Automated methods error on an MRI sagittal slice of a typical subject imaged at 1.5T (left) and 3T (right). Areas of negative error (estimate smaller than the manual reference) are shown in green, areas of positive error in red. Areas identified as intracranial by both the gold standard and the automated method segment are shown in white. Top panel: MR images with isolines of the manual delineation. Second panel: SPM-tissue class method (default setting) output. Third panel: BET output. Bottom panel: RBM output.

The volume of the intracranial portion of the head identified by the established methods was too small to serve as a reasonable approximation of ICV for the images obtained from the 3T scanner (see Results and Fig. 5). We noted a discrepancy in intensities between intraventricular and cisternal CSF at 3T (Fig. 4), a difference that was not seen at 1.5T and could be at the root of the underestimation of ICV on 3T images with SPM5. To investigate whether this intensity variation is field-strength specific rather than scanner-specific, we performed CSF sampling on images from all scanners described in the Subject section. For this procedure, a grey matter–white matter (GMWM) probability map was obtained as the sum of grey matter (GM) and white matter (WM) probability maps produced by SPM5. ICV was measured with the RBM method described above. The brain CSF mask, intraventricular CSF, and CSF in the subarachnoid space were estimated by subtracting the GMWM mask, thresholded at 90%, from the ICV mask. We used the inverted registration of the standard space to the individual subject scan to warp a linearly generated MNI152 template brain mask which is included in FSL. The resulting conservative mask identifies the brain voxels in standard space where the brain was located 50% or more of the time for the aligned subjects used in MNI152. The purpose of this mask was to estimate the intraventricular CSF in an individual scan. A sequence of morphological operations along with the CSF probability map as prior knowledge was used to separate the intraventricular CSF from the CSF in the subarachnoid space. In neuroanatomy, cisterns refer to any of the openings in the subarachnoid space of the brain filled with cerebrospinal fluid. However, some of the major subarachnoid cisterns (e.g., pontine cistern, interpeduncular cistern, ambient cistern, and so on) locate in a central part of the brain. Therefore, what we measured as the “peripheral” CSF is a combination of the peripheral and some parts of central CSF. We used a Monte Carlo simulation and 10 trials sampling of 270 voxels. This sampling size was based on 90% confidence and acceptance of an error of 10% in the CSF intensity standard deviation from intraventricular and cisternal CSF.

### Statistical analysis

The reliability of manual ICV determination was assessed using a test-retest strategy. The same rater determined the intracranial portion twice with an interval of one month. Relative volume differences between the test-retest pair were measured as a percentage:

(1) $${\text{\%DIFF}}_{V_{2}-V_{1}}=\left(\frac{V_{2}-V_{1}}{\left(V_{2}+V_{1}\right)/2}\right)\times 100$$

where *V*_2_ and *V*_1_ are retest and test measurement, respectively.

Although relative volume difference may highlight systematic over- or underestimation, it might mask random (non-systematic) error. For example, a more noisy (and therefore less robust) method might generate values that either over- or underestimate ICV across different subjects. The mean of these measurements can be misleadingly close to zero because underestimations in some subjects will be canceled out by overestimations in others. Therefore, the magnitude of the relative differences was also measured using:

(2) $${\text{\%ADIFF}}_{V_{2}-V_{1}}=\left(\frac{\left|V_{2}-V_{1}\right|}{\left(V_{2}+V_{1}\right)/2}\right)\times 100$$

where |·| is the absolute operator. This measure shows the robustness of a method without any indication of the over- or underestimation of the ICV.

Reliability assessments based on volume differences alone are insufficient, as the measure is insensitive to segmentation errors that compensate for each other (positive error balancing out negative error). An additional measure, the Dice coefficient (Dice, 1945), was therefore used to assess the overlap between the test–retest pair of segmentations. If *N*(*A*), *N*(*B*) and *N*(*A*∩*B*) represent the volumes of ICV measured for two subjects and their intersection, then the Dice coefficient (DC) is defined as:

(3) $$\text{DC}=\frac{2\times N\left(A\cap B\right)}{N\left(A\right)+N\left(B\right)}$$

The result of the ICV measurement using three different methods of bias correction along with different ICV measurement methods, ICV_BET, ICV_SPMA, ICV_SPMB and ICV_RBM was evaluated in terms of the relative volume differences and its magnitude, Dice coefficient and intraclass correlation coefficient (ICC) with the manual ICV measurement (ICV_MANUAL). Statistical analysis was performed using SPSS Version 16 for Microsoft Windows (SPSS Inc., Chicago, IL, USA).

To assess the influence of scanner field strength on the accuracy of ICV measurement, we calculated the positive and negative error for the automated methods as follows. For each manually delineated slice, we defined the false-positive area (or “positive error”) as the area that the automated method incorrectly segmented as intracranial tissue compared to the manually delineated slice. We defined the false-negative area (“negative error”) as the area that the automated tool incorrectly segmented as non-intracranial tissue compared to the manually delineated slice.

## Results

### Manual test–retest reliability

The average relative volume difference from first to second manual measurement of ICV in the five subjects was − 0.4% ± 0.76 (range, − 1.2 − 0.83%) for the images obtained from the 1.5T scanner and − 0.3% ± 0.5 (range − 0.2 − 0.6%) for those scanned with the 3T scanner. Average DC were 0.94 ± 0.01 and 0.96 ± 0.02 for subjects scanned with the 1.5T and 3T scanner, respectively. The ICVs obtained from the 3T scanner were 0.74 ± 0.3% larger than those obtained from 1.5T scanner (paired *t*-test, *p* < 0.001). This implies that they are systematically slightly different even for manual segmentation, but this difference is well within the likely calibration error.

### ICV measurement

**Fig. 6:**
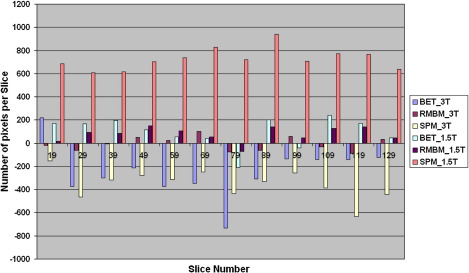
Average positive and negative error by slice (*n* = 12) between manually segmented ICV and BET, SPM-tissue class, and RBM method results (Slice 19 is the left slice in the brain, Slice 129 is the top) in five subjects scanned at 1.5T and 3T.

We performed four different analyses of accuracy based on the five subjects in Group 1:

1. *Relative volume differences and its magnitude between manually and automatically calculated ICV*: As described in 2.7 the relative volume differences and its magnitude between manual measurement and automated ICV measurements were calculated (Tables 1, 2 and 3), where, in Eq. 1, *V*_2_ and *V*_1_ are ICV_MANUAL and ICV measured with automated methods (ICV_BET, ICV_SPMA, ICV_SPMB and ICV_RBM) for each of the bias correction methods (FAST, SPM and N3), respectively. In addition, the magnitude of the relative volume differences were calculated as a measure of robustness.
2. *Overlap between ICV_MANUAL and the other automated methods*: Overlap measures expressed as Dice coefficients (Tables 1, 2 and 3).
3. *Correlation between manually and automatically calculated ICV (ICC)*: Tables 1, 2 and 3 show the degree of correlation between ICV_MANUAL and the automated methods.
4. *Association between scanner field strength and method accuracy*: The measurement of the total false-positive and false-negative error shows that the SPM-tissue class method and BET resulted in more negative error than RBM on the images obtained from the 3T scanner (Fig. 5 (d,f)), and the SPM-tissue class method had more positive error than RBM in images obtained at 1.5T (Figs. 5, 6).

Table 1 shows that FAST+SPM (A and B) in comparison with FAST+BET or FAST+RBM does not perform well and the resulting ICVs are smaller than the gold standard in images obtained from the 3T scanner. Both FAST+RBM and FAST+BET perform well on 3T images. However, the performance of FAST+BET is not as good as FAST+RBM in the case of the 1.5T scanner.

When SPM is used for bias correction, it can be seen that in the 3T scanner SPMB performs better than SPMA in computing ICV, while in the 1.5T scanner SPMA yields better results than SPMB (Table 2). The results obtained by BET and RBM methods in both scanners are comparable, but the relative volume difference and its magnitude is smaller for RBM than for BET in the 1.5T scanner.

When N3 is used for bias correction, ICV results on 1.5T images are too large, while ICV results on 3T images are closer to the manual reference. Table 3 shows that the N3+RBM pipeline performs nominally better than any other pipeline.

Tables 1, 2 and 3 show that RBM is the most robust and accurate method for the ICV measurement for images obtained from scanners with different field strengths, regardless of which bias correction method is used. Using automated methods (SPM or BET) to estimate ICV yielded acceptable intraclass correlation coefficients between 1.5T and 3T images, but both show a larger systematic bias (relative volume difference) than the RBM method. Both SPMA and SPMB consistently overestimated ICV on 1.5T images and underestimated ICV on images obtained from 3T scanners. When BET was used, this systematic bias was inverted.

The relative volume difference, its magnitude and DC were calculated between manual measurement and automated ICV measurements for two subjects in Group 2, after applying SPM's bias correction method (Table 4). Table 4 confirms that RBM is the most robust and accurate method for ICV measurement even in patients with AD for images obtained from scanners with different field strengths. When different ICV measurement methods were applied to all the subjects in Group 2, RBM was more consistent between different field strengths (ICC = 0.97) in comparison with the other methods, BET (ICC = 0.96), SPMA (ICC = 0.65) and SPMB (ICC = 0.7).

For further analysis, we used RBM to obtain ICV measurements on images of Group 4. Measurements obtained on 1.5T images tended to be smaller compared to 3T images (relative difference − 0.1 ± 2.5%).

### Influence of field strength on ICV measurement

The intensity of intraventricular and cisternal CSF was measured with the sampling method described above (see Section 2.6) in the 10 MR scans of Group 1. The average CSF intensity difference between intraventricular and cisternal CSF was 16 ± 60% in images obtained at 1.5T and 32 ± 54% in images obtained at 3T, with higher intensity in the intraventricular region than in the cisternal region before applying bias correction. Means, standard deviations, and ranges of the intraventricular and cisternal CSF intensity difference were calculated for all images after application of the different non-uniformity correction methods (FAST, SPM5 and N3) (Table 5). FAST and SPM bias correction achieved greater uniformity of CSF signal than N3. These demonstrated greater uniformity for corrected 1.5T images (smaller relative difference) than for 3T images.

**Fig. 7:**
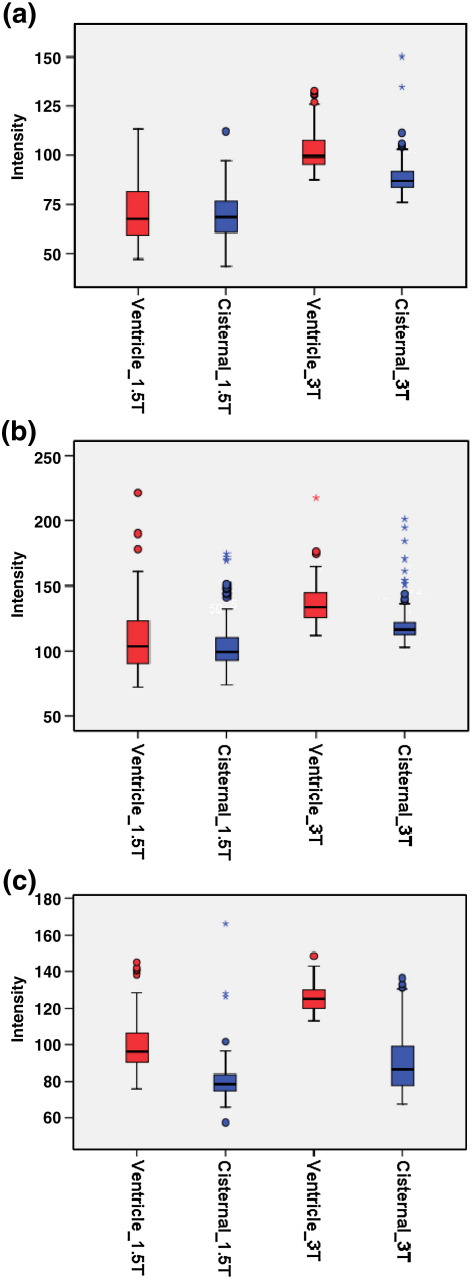
Average CSF intensity of intraventricular and cisternal CSF in five subjects (10 MRIs) obtained at 1.5T and 3T with different bias correction methods. (a) FAST, (b) SPM, (c) N3. Horizontal lines: median; boxes: interquartile ranges; whiskers: range; circle: outlier. Blue: cisternal; red: intraventricular CSF intensity. For each scanner, average CSF intensity in ventricle is shown first, followed by the average cisternal CSF intensity.

Figure 7 shows that none of the bias correction methods was able to eliminate the problem of CSF intensity in the cisternal region being lower than that in the intra-ventricular region in images obtained at 3T.

N3′s performance was poor in reducing the CSF intensity difference between the abovementioned regions as shown in Table 5. The imperfect result of N3 in comparison with the other two methods may have been a consequence of applying this method on the full volume instead of a skull-stripped image. SPM and FAST were able to reduce this discrepancy to an acceptable level in 1.5T images, but for 3T images, the methods were also unsuccessful.

**Fig. 8:**
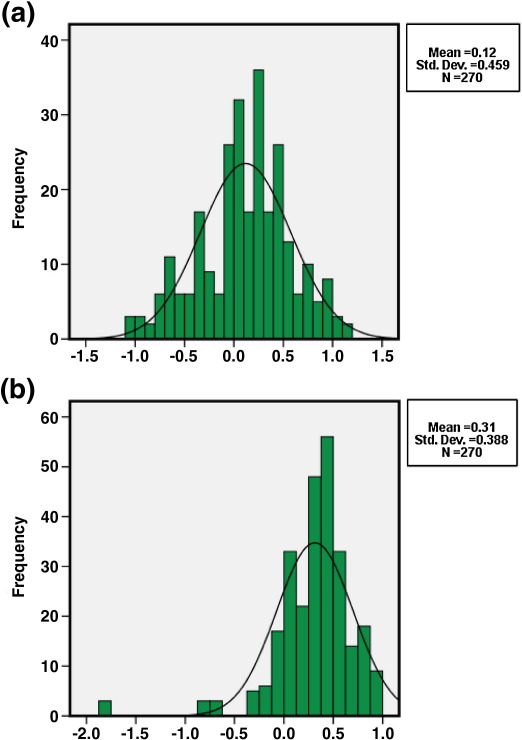
Histograms of the subtraction distribution for a single subject scanned with (a) 1.5T and (b) 3T.

Both the intraventricular and cisternal CSF of 3T and 1.5T scanners showed Gaussian distributions, somewhat skewed, which could be substantially normalized by using the log transform. Therefore, log intensity was used for determining the difference between intraventricular and cisternal CSF in images obtained at different field strengths. A Kolmogorov–Smirnov test (KS) indicated that the intensity difference between intraventricular and cisternal CSF measures within 1.5T scanners was normally distributed (*p* > 0.1), whereas this intensity difference measured at 3T was significantly different from a normal distribution (*p* < 0.05) (Fig. 8).

It is conceivable that the CSF intensity difference we found could be due to the specific scanners, coils and acquisition parameters used in Group 1. We therefore performed the CSF sampling method on the subjects of Group 3 and Group 4 obtained with different scanners. On average, the CSF intensity in the intraventricular region was 11 ± 9% higher than CSF intensity in the cisternal region after applying SPM bias correction to images obtained at 3T in Group 3, while the observed intensity difference in 1.5T images was smaller, 2 ± 16%. In Group 4, even after the multi-step pre-processing corrections applied as part of the ADNI pre-processing (Gradwarp, B1 correction and using N3), while this difference was not consistent, three out of five subjects showed the abovementioned difference. However, the relative difference was smaller than for subjects in Group 1 and Group 3. For subjects in Group 4, the observed intensity difference in 1.5T images was 2 ± 4% and for images obtained at 3T this difference was 7 ± 5%.

The total CSF volume of the brain, combination of the CSF in the intraventricular and the subarachnoid spaces, and the cisternal CSF volume were estimated with the method described in Section 2.6 in the 10 MR scans of Group 1. The total CSF volume obtained from the 1.5T scanner was 14.3 ± 6.2% larger than those obtained from the 3T scanner. Furthermore, the estimated volume of the cisternal CSF obtained from 1.5T images was 16.8 ± 7.9% larger compared to 3T images. This difference implies systematic differences in ICV measurements, particularly peripherally using established methods. Means and standard deviations, of the total, intraventricular and cisternal CSF volume were calculated for all images (Table 6).

## Discussion

Intracranial volume has been recognized as a suitable constant for normalizing the size of individual brain structures, (Free et al., 1995; Eritaia et al., 2000). Compared to other commonly used constants, in particular total brain volume, ICV is less vulnerable to pathological changes. In this work, we used MR images from scanners with two types of field strength (1.5T and 3T) to address the important issue of differences in ICV estimates that are attributable to the difference in field strengths. We found that ICV measurements using established methods are not comparable, and that the estimated intracranial portion of 3T images typically excludes cisternal CSF due to systematically different intensities between cisternal and intraventricular fluid spaces. We showed that the cisternal CSF space that surrounds the brain (subarachnoid space) showed intensity values close to those of CSF inside the brain (ventricular system) in images obtained at 1.5T, while the CSF intensity in these two areas was different in 3T images. Some of the subarachnoid cisternal CSF is located centrally in the image space, but we only distinguish between ventricular and cisternal CSF. Our measurement is therefore likely to underestimate the real intensity difference between these locations.

The phenomenon appears to be closely related to central brightening artifacts which manifest themselves as high signal intensity in the center of head images. It is not unusual to observe central brightening of 30% at 3T, compared with 5% at 1.5T (Bernstein et al., 2006). The central brightening effect can be reduced when using array coils for signal reception (Bernstein et al., 2006). Most commonly post-processing methods are applied to achieve intensity nonuniformity correction (bias correction) using methods such as N3 (Sled et al., 1998), SPM (Ashburner and Friston, 2005) or FSL (FAST) (Zhang et al., 2001). Although receiver array coils was used in Groups 1 and 2 and we tried three different bias correction algorithms in this study, we still observed the effect of this phenomenon on the 3T images. Bias correction methods simply scale the acquired signals to achieve a more uniform intensity distribution, but do not attempt to improve contrast between adjacent tissues. An issue with the 3T data we investigated is that some tissues, specifically CSF and dura mater, have signal intensities that are similar.

A number of other studies have contrasted the performance of different bias correction methods and their effects on voxel-based morphometry (VBM) (Acosta-Cabronero et al., 2008) or tissue segmentation (Clark et al., 2006; Arnold et al., 2001). However, most of the studies were based on 1.5T scanners and none of them examined and compared scanners with different field strengths.

We found that, with common tissue segmentation techniques such as SPM5 on T1-weighted images, a considerable portion of CSF may be excluded and this constitutes a source of systematic error in the ICV estimation. We applied different settings based on several studies that compared different automated ICV measurement methods (Acosta-Cabronero et al., 2008; Hartley et al., 2006; Pengas et al., 2009). Pengas et al. (2009) reported that either proton density (PD) or T2-weighted images were least susceptible to atrophy in semantic dementia in the ICV estimation. However, there are two reasons which limit the use of these methods for measuring intracranial volume: (i) most MR image processing methods developed so far are based on the T1-weighted sequence; (ii) PD and T2-weighted images are not available in all databases, and the T1-weighted sequence is the most commonly available image acquisition, particularly when MRI was mainly acquired to provide higher anatomical resolution for co-registration with PET or SPECT images. The comparisons of conventional methods for estimating ICV showed that none of them yielded consistent results across different scanners. For example, the intracranial mask could extend into dura mater and bone, include the orbital fat or eyeballs or exclude portions of cerebellum.

Reverse template brain masking methods have been used in other studies of data acquired at one field strength only (Ishii et al., 2006; Quarantelli et al., 2002; Driscoll et al., 2009; Resnick et al., 2009). To address the problems described in this study, we propose an automated measure of ICV using a standard mask in MNI space derived from MNI's tissue probability maps, and inverse transformations to warp the standard-space brain mask to each image in native space. Automated measures of ICV with the proposed method are highly consistent with manual total intracranial volume (ICC = 0.99).

The delineation of the intracranial portion of a brain image as provided by the RBM method has two uses in MR images analysis. The first use is as an efficient, robust estimate of ICV for serving as a covariate or normalization factor in morphometric analyses of regional and whole brain volumes, much in the same manner as a manually measured ICV but obtained rapidly and without user intervention. In addition, this method has the potential to be used as a skull stripping method during pre-processing for image registration in brain morphometry (Heckemann et al., 2006).

## Figures and Tables

**Table 1:** Relative (DIFF), magnitude (ADIFF) volume difference, DC (Dice coefficient) and ICC using FAST bias correction along with four ICV measurement methods. Positive differences mean that the manual estimation was larger.

	FAST-SPMA	FAST-SPMB	FAST-BET	FAST-RBM
*1.5T*				
%DIFF	− 3.0 ± 8.8	− 5.0 ± 9.3	− 4.2 ± 2.4	− 0.9 ± 3.3
%ADIFF	6.5 ± 6.1	8.0 ± 6.1	4.2 ± 2.4	2.1 ± 1.7
DC	0.88 ± 0.02	0.88 ± 0.02	0.91 ± 0.02	0.92 ± 0.01
ICC	0.88	0.86	0.95	0.98

*3T*				
%DIFF	9.5 ± 2.0	9.5 ± 1.7	0.5 ± 2.4	0.4 ± 1.7
%ADIFF	9.5 ± 2.0	9.5 ± 1.7	2.1 ± 0.9	0.7 ± 1.3
DC	0.84 ± 0.01	0.84 ± 0.01	0.93 ± 0.02	0.94 ± 0.01
ICC	0.82	0.83	0.99	0.99

**Table 2:** Relative (DIFF), magnitude (ADIFF) volume difference, DC (Dice coefficient) and ICC using SPM bias correction along with four ICV measurement methods. Positive differences mean that the manual estimation was larger.

	SPM-SPMA	SPM-SPMB	SPM-BET	SPM-RBM
*1.5T*				
%DIFF	− 4.5 ± 7.4	− 12.4 ± 7.2	− 2.9 ± 1.9	− 0.7 ± 1.4
%ADIFF	7.1 ± 4.0	12.4 ± 7.2	2.9 ± 1.9	1.2 ± 0.8
DC	0.87 ± 0.01	0.89 ± 0.01	0.91 ± 0.02	0.91 ± 0.01
ICC	0.81	0.67	0.98	0.98

*3T*				
%DIFF	10.2 ± 1.3	3.0 ± 2.5	1.7 ± 2.1	1.6 ± 1.1
%ADIFF	10.2 ± 1.3	3.3 ± 1.6	1.7 ± 2.1	1.6 ± 1.1
DC	0.90 ± 0.01	0.84 ± 0.03	0.93 ± 0.02	0.93 ± 0.01
ICC	0.73	0.97	0.98	0.99

**Table 3:** Relative (DIFF), magnitude (ADIFF), DC (Dice coefficient) and ICC using N3 bias correction along with four ICV measurement methods. Positive differences mean that the manual estimation was larger.

	N3-SPMA	N3-SPMB	N3-BET	N3-RBM
*1.5T*				
%DIFF	− 11.4 ± 8.2	− 11.0 ± 8.0	− 3.2 ± 2.3	− 0.8 ± 3.6
%ADIFF	11.4 ± 8.2	11.0 ± 8.0	3.2 ± 2.3	2.0 ± 1.9
DC	0.70 ± 0.04	0.72 ± 0.03	0.91 ± 0.02	0.91 ± 0.01
ICC	0.48	0.50	0.97	0.98

*3T*				
%DIFF	0.3 ± 3.8	0.4 ± 3.3	1.6 ± 2.4	− 0.08 ± 1.7
%ADIFF	2.9 ± 1.9	2.3 ± 2.1	2.2 ± 1.8	0.5 ± 0.7
DC	0.92 ± 0.02	0.91 ± 0.02	0.93 ± 0.02	0.94 ± 0.01
ICC	0.98	0.98	0.98	0.99

**Table 4:** Relative (DIFF), magnitude (ADIFF) volume difference with manual ground truth, DC (Dice coefficient) and ICC using four ICV measurement methods for two subjects with AD. Positive differences mean that the manual estimation was larger.

	SPMA	SPMB	BET	RBM
*1.5T*				
%DIFF	− 2.4 ± 5.5	− 2.5 ± 5.6	− 0.2 ± 2.3	− 0.2 ± 0.09
%ADIFF	3.9 ± 3.4	3.9 ± 3.5	1.6 ± 0.3	0.2 ± 0.09
DC	0.88 ± 0.01	0.89 ± 0.03	0.90 ± 0.01	0.94 ± 0.03

*3T*				
%DIFF	4.3 ± 0.5	− 0.2 ± 6.7	1.1 ± 0.6	− 0.5 ± 0.2
%ADIFF	4.3 ± 0.5	4.7 ± 0.3	1.1 ± 0.6	0.5 ± 0.2
DC	0.81 ± 0.03	0.81 ± 0.02	0.88 ± 0.02	0.95 ± 0.01

**Table 5:** Descriptive statistics of intra-ventricular and cisternal CSF intensity difference in Group 1 for three bias correction methods. Positive values indicate higher intensity in the intraventricular CSF.

Scanner	FAST mean ± SD range	SPM5 mean ± SD range	N3 mean ± SD range
1.5T	1 ± 18	3 ± 18	10 ± 12
− 54–44	− 40–67	− 51–60
3T	14 ± 12	12 ± 15	26 ± 16
− 38–46	− 28–63	− 10–66

%Differences of *V*_2_–*V*_1_, [(*V*_2_ − *V*_1_)/((*V*_2 _+ *V*_1_) / 2)] × 100:*V*_1_ = cisternal CSF intensity and *V*_2_ = ventricular CSF intensity.

**Table 6:** Descriptive statistics of total, intra-ventricular and cisternal CSF volumes in Group 1.

Scanner	Total CSF mean ± SD	Intra-ventricular CSF mean ± SD	Cisternal CSF mean ± SD
1.5T	276.1 ± 125.3	312.9 ± 21.8	244.8 ± 98.8
3T	237.3 ± 105.7	318.6 ± 24.3	205.4 ± 95.4

Values are described as the volume in units of cm^3^.

## Footnotes

2 www.adni-info.org, www.ixi.org.uk, www.neurogrid.ac.uk

3 The ADNI was launched in 2003 by the National Institute on Aging (NIA), the National Institute of Biomedical Imaging and Bioengineering (NIBIB), the Food and Drug Administration (FDA), private pharmaceutical companies and non-profit organizations, as a $60 million, 5-year public-private partnership. The Principal Investigator of this initiative is Michael W. Weiner, M.D., VA Medical Center and University of California-San Francisco. ADNI is the result of efforts of many co-investigators from a broad range of academic institutions and private corporations, and subjects have been recruited from over 50 sites across the United States and Canada.

4 Since a tissue probability map may be shared by several clusters, the number of Gaussians used to represent the intensity distribution for each tissue classes can be greater than one. Default numbers of Gaussians used in SPM5 are two for grey matter (GM), two for white matter (WM), two for CSF, and four for everything else (Ashburner and Friston, 2005). We hypothesized that because of the intensity differences in intraventricular and cisternal CSF we found in 3T scanner data, the CSF intensity would be too variable to be well modeled by only two Gaussians.

5 www.fmrib.ox.ac.uk/fsl

6 www.loni.ucla.edu
